# Quit Now? Quit Soon? Quit When You’re Ready? Insights About Target Quit Dates for Smoking Cessation From an Online Quit Date Tool

**DOI:** 10.2196/jmir.3086

**Published:** 2014-02-17

**Authors:** Caroline O Cobb, Raymond S Niaura, Elisabeth A Donaldson, Amanda L Graham

**Affiliations:** ^1^Schroeder Institute for Tobacco Research and Policy StudiesLegacyWashington, DCUnited States; ^2^Department of Health, Behavior and SocietyJohns Hopkins Bloomberg School of Public HealthBaltimore, MDUnited States; ^3^Department of Oncology, Georgetown University Medical Center / Cancer Prevention and Control Program, Lombardi Comprehensive Cancer CenterWashington, DCUnited States

**Keywords:** smoking cessation, Internet, quit date, tobacco dependence

## Abstract

**Background:**

Setting a target quit date (TQD) is often an important component in smoking cessation treatment, but ambiguity remains concerning the optimal timing (ie, quitting spontaneously versus delaying to prepare).

**Objective:**

We examined four questions about the timing of TQDs and smoking outcomes in secondary analyses of The iQUITT Study, a randomized trial of Internet and telephone treatment for cessation: (1) What are the characteristics of TQDs set using an online interactive quit date tool?, (2) What are the characteristics of individuals who use a quit date tool and do they differ from those who do not?, (3) Are there differences in smoker characteristics, treatment utilization, and cessation outcomes based TQD timing?, and (4) Is maintenance of an initial TQD predictive of abstinence or do changes to TQDs lead to cessation?

**Methods:**

A total of 825 adult current cigarette smokers were randomized to enhanced Internet or enhanced Internet plus telephone counseling. Latency to TQD in days was calculated as the date difference between the initial TQD and enhanced Internet registration; prospective TQD setters were stratified into four latency groups (0, 1-14, 15-28, 29+ days). Baseline variables, website utilization, and 3-month cessation outcomes were examined between prospective TQD groups. Desire and confidence to quit, number of TQDs, and website logins were tested as predictors of 30-day point prevalence abstinence (ppa) at 3 months (responder-only analyses). Classification and regression tree (CART) analysis explored interactions among baseline variables, website utilization, and latency to TQD as predictors of 30-day ppa.

**Results:**

There were few baseline differences between individuals who used the quit date tool and those who did not. Prospective TQDs were set as follows: registration day was 17.1% (73/427), 1-14 days was 37.7% (161/427), 15-28 days was 18.5% (79/427), and 29+ days was 26.7% (114/427). Participants with a TQD within 2 weeks had higher baseline self-efficacy scores but did not differ on smoking variables. Individuals whose TQD was the same day as registration had the highest logins, page views, number of TQDs set using the tool, and messages sent to other members. Logistic regression revealed a significant interaction between number of TQDs and website logins for 30-day ppa (*P*=.005). Among those with high logins, 41.8% (33/79) with 1 TQD were abstinent versus 25.9% (35/135) with 2+TQDs. Logins and self-efficacy predicted 30-day ppa in the CART model.

**Conclusions:**

TQD timing did not predict cessation outcomes in standard or exploratory analyses. Self-efficacy and an apparent commitment to an initial TQD were the components most highly related to abstinence but only via interactions with website utilization. Findings highlight the importance of feeling efficacious about handling specific smoking situations and engaging with treatment. Additional research focused on increasing engagement in Web-based cessation studies is needed.

**Trial Registration:**

ClinicalTrials.gov: NCT00282009; http://clinicaltrials.gov/show/NCT00282009 (Archived by WebCite at http://www.webcitation.org/6Kt7NrXDl).

## Introduction

Setting a quit date is often a central element of tobacco dependence treatment [1-4]. Establishing a target quit date (TQD) may increase the likelihood of success for several reasons. The public commitment often involved in setting a quit date may increase or solidify a smoker’s motivation to quit [5] and the probability that they will follow through with intentions to quit [6]. Setting a TQD may also provide time for the smoker to develop relevant coping skills [6,7] and to obtain and initiate medication use, which can increase the likelihood of abstinence [2,8].

However, there is mixed evidence regarding the importance of the nature (ie, planned vs unplanned) and timing (ie, sooner vs later) of quit dates. Some evidence suggests that setting a TQD is associated with a greater likelihood of making a quit attempt [9] and is a predictor of abstinence [10,11]. Other studies indicate that roughly half of smokers prefer to quit abruptly [12] and do not plan a quit attempt [13-16] and that unplanned or spontaneous quit attempts are more likely to be successful than those involving a TQD [13-17]. It is also unclear whether the timing of a quit date matters. A recent randomized controlled trial by Hughes et al [18] in which smokers were prompted to select a quit date found that those who selected a later quit date or delayed a planned quit attempt were less likely to quit smoking compared to participants who selected an early quit date or adhered to their original date. Similarly, in a trial of varenicline versus placebo for smoking cessation in which smokers chose their own quit dates (within a 5-week time frame), smokers who selected later quit dates (particularly in the last week) were less likely to achieve abstinence in both treatment arms [19]. In contrast, among smokers who planned to quit within a month, proximity of the quit date did not predict abstinence [9]. Similarly, in a Web-based trial by Etter et al [12], smokers randomized to abrupt versus gradual quitting had equivalent quit rates at all follow-ups.

This ambiguity regarding quit dates is reflected in the varying recommendations found on smoking cessation websites. For example, the instructions on the American Cancer Society’s website state “Once you’ve decided to quit, you’re ready to pick a quit date. This is a very important step. Pick a day within the next month as your Quit Day” [20]. The American Legacy Foundation’s BecomeAnEX website instructs smokers “Don’t pick tomorrow as your quit date… Don’t set your date too far off in the future… We recommend a day that’s about 2-4 weeks away” [21]. The National Cancer Institute’s cessation website tells smokers who are preparing to quit to “Pick a date within the next 2 weeks to quit” [22]. QuitAssist, a free website provided by the tobacco company, Altria, simply encourages smokers to “get ready” and “choose a specific quit date” with no specific timeline [23]. For the millions of smokers who search online for assistance quitting smoking [24-26], these mixed messages may be confusing.

Most studies that have examined the timing of a quit date have used retrospective, cross-sectional population-based survey data [13-17] or data gathered in the context of randomized controlled trials in which participants were required to set a quit date or adhere to a researcher-defined date [27,28]. Each of these approaches has limitations. Retrospective reports are subject to recall bias skewed toward remembering more planned quit attempts [29], and required quit dates may not be representative of actual quitting behavior. Prospective research is needed that uses objective methods for measuring the timing of quit dates that occur naturally during the course of quitting.

Web-based cessation programs represent both an effective intervention approach to help smokers quit and a means to address some of the limitations of previous analyses of quitting behavior. Sites that offer interactive tools to assist users in choosing and/or documenting a quit date [30] can yield prospective, naturalistic, and objective measures of quitting behavior with regard to the nature and timing of quit dates. We are aware of only one study that has examined the use of an online quit date tool and its association with abstinence [31].

Our study examined four key questions: (1) What are the characteristics of quit dates that are set using an online interactive quit date tool?, (2) What are the characteristics of individuals who use a quit date tool and do they differ from those who do not?, (3) Are there differences in smoker characteristics, treatment utilization, and cessation outcomes based on the timing of an initial (ie, first) TQD in relation to program initiation?, and (4) Is the maintenance of a TQD predictive of eventual abstinence, or are multiple changes of an online quit date more likely to lead to cessation? We approached these questions in secondary analyses of data from a pragmatic randomized trial of Internet and telephone treatment for smoking cessation [32]. Participants were not required to set a quit date and could use the website as they desired. We began with standard analytic methods to describe differences among those who used an online interactive quit date tool and those who did not. We then examined differences among prospective quit date setters based upon the latency to an initial TQD. We hypothesized that individuals whose target quit date was within 2 weeks of registration would be more motivated to quit, have higher indices of treatment utilization, and be more likely to maintain abstinence. We also hypothesized an interaction between the number of TQDs set and website utilization, such that the highest abstinence rates would be observed among participants with only one TQD (signaling unwavering commitment) and high levels of website utilization. To guide future studies, we employed an exploratory data analysis technique, classification and regression tree analysis (CART) [33], to examine the interactive nature of various predictors on abstinence. This exploratory method can augment traditional analytic approaches to identify unique combinations of variables related to tobacco use behavior patterns [34,35].

## Methods

### Participants

Participants in The iQUITT Study [32,36] were smokers aged 18 and older in the United States who smoked 5 or more cigarettes per day. To maximize generalizability of study findings, motivation to quit and willingness to set a quit date were not included as eligibility criteria. Active user interception sampling was used to recruit smokers who used the terms “quit(ting) smoking”, “stop(ping) smoking”, or “smoking” in a major Internet search engine and who clicked on a link to QuitNet, the cessation treatment website being evaluated [37]. Following online informed consent and a baseline telephone assessment, participants were randomized to basic Internet, enhanced Internet, or enhanced Internet plus telephone counseling in the parent trial. Follow-up assessments were conducted by phone or online for telephone non-responders at 3, 6, 12, and 18 months. These analyses focus on participants with complete 3-month follow-up data in the two enhanced Internet arms, which included an interactive tool to assist users in setting a quit date (“Quit Date Wizard”). The basic Internet intervention did not include the Quit Date Wizard. Across both enhanced Internet arms, 75% (995/1326) of participants were reached at 3 months. Due to a technical issue early in the trial, data on use of the Quit Date Wizard were not stored for 170 participants. Thus, the final sample for these analyses focused on 825 participants (412 enhanced Internet, 413 enhanced Internet plus telephone counseling).

### Interventions

Participants randomized to enhanced Internet were given 6 months of free access to the premium service of the QuitNet website. QuitNet is a widely used Internet cessation program that incorporates evidence-based elements of tobacco dependence treatment [2] including practical counseling and tailored information for cessation, recommendations and support for approved pharmacotherapy, and intra-treatment social support through a large online social network [36,38,39].

The Quit Date Wizard is a central feature of QuitNet. It explains the importance of setting a quit date and prompts users to think about a realistic time frame for quitting (“To choose a timeframe, think about approximately when you will be ready to quit”) with options ranging from “In a week” to “In more than 2 months”. The Wizard also encourages users to consider potential triggers, steps to prepare to quit, and pharmacotherapy use. The Quit Date Wizard does not specify an optimal timeframe for quitting but encourages users to consider whether they feel prepared and if not “to spend a few weeks getting to the point where you are comfortable with the idea of ‘jumping in’ [to quitting]”. Users can enter their own date or select a Wizard-generated quit date. Users can also make their quit date visible to other members for support and can sign up for quit support emails timed around their quit date. Repeated reminders to set a quit date using the Quit Date Wizard or to confirm a previously set quit date are featured prominently throughout QuitNet. Users can update their quit date at any time. These analyses focus on the initial TQD, measured as the number of days between website registration and the first TQD that the user set in the Quit Date Wizard. We elected to examine this TQD versus subsequent updates or changes to a quit date to inform recommendations provided by Internet smoking cessation programs. These analyses are not designed to address the timing of a quit date subsequent to a slip or relapse.

Participants randomized to enhanced Internet plus telephone counseling were offered 5 calls in a relapse-sensitive schedule [40]. Counselors had real-time access to summary data regarding a participant’s use of the QuitNet site, which enabled them to prompt and reinforce use of QuitNet (including the Quit Date Wizard) during each call.

### Data Collection and Measures

#### Summary

The three sources of data are described below. These analyses focus on 3-month data since study questions addressed initial quitting behavior, and this is typically where treatment utilization and intervention effects are the strongest.

#### Baseline Assessment

Age, gender, race, ethnicity, education, employment, and household income were assessed. We also assessed self-rated health status [41], history of smoking-related illness, body mass index, and whether they had spoken to a doctor about their smoking. Smoking variables included cigarettes per day, the time to first cigarette item from the Fagerström Test for Nicotine Dependence [42], duration of last quit attempt (days), desire to quit and confidence in quitting (scale=1-10), spouse smoking status, and number of smokers in the home. Psychosocial items included the Smoking Situations Confidence Inventory and the Smoking Temptations Inventory (short-form) [43] as measures of self-efficacy, the Perceived Stress Scale [44], the Center for Epidemiologic Studies-Depression (CES-D) Scale [45], Weight Concern Scale [46], the Social Network Index [47], an abbreviated version of the Partner Interaction Questionnaire [48,49], and an item from the Two-Item Conjoint Screen [50] assessing alcohol consumption.

#### Three-Month Follow-Up Assessment

Smoking outcomes included number of intentional quit attempts and 30-day point prevalence abstinence (ppa; primary outcome of the parent trial) calculated using responder-only analyses. Participants also reported use of other quit methods since enrolling in the trial, including nicotine replacement therapy, behavioral treatment (eg, self-help materials, individual counseling), and prescription medication use (eg, bupropion).

#### Treatment Utilization

Website utilization metrics included date of QuitNet registration, date of initial TQD, total number of quit dates set using the Quit Date Wizard, website logins, page views, total time online, exchange of messages with other QuitNet members (yes/no), and use of an interactive Medication Wizard (yes/no). Number of calls completed was examined among individuals randomized to enhanced Internet plus telephone counseling.

### Statistical Analyses

For Study Question 1, frequency counts were used to characterize use of the Quit Date Wizard. Latency to TQD (days) was calculated as the difference between the first date designated using the Quit Date Wizard and the website registration date. To anchor our analyses to common recommendations provided to smokers in Web-based cessation programs, we categorized this variable as 0 days (registration day), 1-14 days (within 2 weeks), 15-28 days (2-4 weeks), and 29+ days (more than 4 weeks). For Study Question 2, selected baseline characteristics of QuitNet registrants were compared between those who set a quit date using the Quit Date Wizard and those who never set a quit date. For Study Question 3, selected baseline characteristics, treatment utilization metrics, and smoking outcomes were examined by latency to TQD using the categories described above: 0 days, 1-14 days, 15-28 days, and 29+ days. We report the median and interquartile range for skewed variables. Between-group comparisons of categorical items and skewed variables were analyzed using nonparametric statistics, and continuous items were analyzed with analysis of variance (ANOVA) using IBM SPSS (version 21.0). For Study Question 4, a logistic regression model examined 30-day ppa as the primary outcome, number of quit dates set using the Quit Date Wizard, number of logins, and the interaction term (centered at the mean) as predictors, and treatment group, desire to quit, and confidence in quitting as covariates using JMP (version 10.02). We examined Study Questions 1-3 by treatment arm and found no between group differences on likelihood of use of the Quit Date Wizard, latency to TQD, baseline characteristics, or website utilization metrics. Therefore, we combined participants from both treatment arms and report the results for the combined sample.

Classification and regression trees (CART) analysis was performed in JMP (version 10.02) to explore the effects of study condition, all baseline variables, and selected treatment utilization measures (logins, number of quit dates set using the Quit Date Wizard, Medication Wizard use, latency to TQD, behavioral treatment use, and pharmacotherapy use) on 30-day ppa, the main outcome of the parent trial [32]. CART analysis allows for a flexible format in terms of allowable response and predictor variables, and handling of missing data [33]. CART is a machine-learning approach that utilizes a classification algorithm to split data into binary subgroups (branches) based upon predictor variables in order to maximize the homogeneity of the two samples for the outcome of interest. In JMP, binary splits for a categorical dependent variable (Y) like abstinence (yes, no) are determined by maximizing the LogWorth statistic ((-log10(*P* value)) [51]. The factors (X; predictors) can be either continuous or categorical (nominal or ordinal). If X is continuous, then the partition is done according to a splitting “cut” value for X. If X is categorical, then it divides the X categories into two groups of levels and considers all possible groupings in two levels. Our CART model included all predictor variables entered simultaneously. To gauge the reliability of our CART analyses, we utilized a k-fold cross-validation procedure that divides the data into k subsets (in this case k=5) that are used to validate the model fit on the rest of the data, fitting a total of K models. The model giving the best validation statistic (-2LogLikelihood) is chosen as the final model.

## Results

### Study Question 1

**Figure 1:**
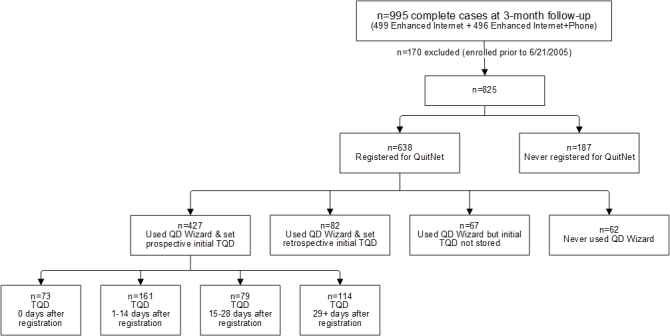
Diagram of data flow from complete cases randomized to the enhanced Internet and enhanced Internet plus telephone counseling arms based on QuitNet registration status, Quit Date (QD) Wizard use, and initial target quit date (TQD) status, and latency to TQD relative to registration date (among prospective quit date setters).

Among all participants, 77.3% (638/825) registered on QuitNet following randomization and 22.7% (187/825) never registered (Figure 1). Among QuitNet registrants, 66.9% (427/638) used the Quit Date Wizard to prospectively set a TQD, 12.9% (82/638) used it to record a TQD that occurred prior to registration (retrospective), and 9.7% (62/638) did not use the tool at all. For 10.5% (67/638) of registered participants, use of the Quit Date Wizard was documented but TQDs were not stored due to a database error.

### Study Question 2

Compared to those who used the Quit Date Wizard (n=576), those who did not (n=62) were more likely to be male (67.7%, 42/62 vs 49.3%, 284/576, *P*=.009) and to have a spouse who smokes (64.9%, 24/37 vs 45.5%, 157/345, *P*=.039). There were no differences on smoking variables, including smoking rate, desire to quit, or confidence in quitting (Multimedia Appendix 1).

### Study Question 3

Among those who set a prospective TQD (n=427), 17.1% (73/427) picked the same day as registration, 37.7% (161/427) picked a date 1-14 days later, 18.5% (79/427) picked a date 15-28 days later, and 26.7% (114/427) picked a date 29 or more days later (see Figure 1). There were differences between prospective TQD groups on education (*P*=.040) and the Confidence Inventory (*P*=.045) (Table 1). There were also differences between groups on treatment utilization metrics. Individuals whose TQD was the same day as registration had the highest number of logins, viewed more webpages, and set more TQDs using the Quit Date Wizard relative to other groups. This group was also the most likely to use one-to-one messaging (31.5%, 23/73) and the least likely to use the Medication Wizard (16.4%, 12/73). Among those who reported at least one quit attempt at the 3-month follow-up (total 389/425; 2 missing cases), there were no differences in use of behavioral quit methods, pharmacotherapy, or telephone counseling calls completed based upon latency to TQD. There were no differences based on latency to TQD on cessation outcomes (Table 2). Overall, 30-day ppa was 21.1% (90/426), and 91.5% (389/425) reported at least one quit attempt.

**Table 1:** Baseline characteristics by latency to target quit date (TQD) relative to website registration date.

Baseline variable			TQD, 0 days	TQD, 1-14 days	TQD, 15-28 days	TQD, 29+ days	*P* value^a^
			n=73	n=161	n=79	n=114	
**Demographic variables** ^b^								
	Age, (years), mean (SD)		34.22 (10.37)	36.99 (10.97)	36.90 (9.98)	38.61 (11.49)	.064
	Gender (Female), n (%)		36 (49.3)	84 (52.2)	37 (46.8)	53 (46.5)	
	**Race, n (%)**						.328
		White	66 (90.4)	145 (90.1)	70 (88.6)	95 (83.3)	
		Non-white	7 (9.6)	16 (9.9)	9 (11.4)	19 (16.7)	
	Ethnicity (Hispanic), n (%)		1 (1.4)	8 (5.0)	3 (3.8)	2 (1.8)	.363
	**Education, n (%)**						.040
		High school or less	9 (12.3)	33 (20.5)	22 (27.8)	37 (32.5)	
		Some college	42 (57.5)	76 (47.2)	34 (43.0)	43 (37.7)	
		College 4+ yrs	22 (30.1)	52 (32.3)	23 (29.1)	34 (29.8)	
	**Employment, n (%)**						.616
		Employed fulltime	50 (68.5)	114 (70.8)	55 (69.6)	87 (76.3)	
		Other^c^	23 (31.5)	47 (29.2)	24 (30.4)	27 (23.7)	
	**Income, n (%)**						.409
		Low income (≤$40,000)	33 (45.2)	69 (42.9)	36 (47.4)	60 (53.1)	
		High income (>$40,000)	40 (54.8)	92 (57.1)	40 (52.6)	53 (46.9)	
**Smoking variables**								
	Cigarettes per day, mean (SD)		20.26 (10.15)	18.75 (7.90)	20.80 (9.32)	19.61 (9.32)	.356
	**Time to first cigarette, n (%)**						.825
		Within 30 minutes	57 (78.1)	122 (75.8)	57 (72.2)	88 (77.2)	
		After 30 minutes	16 (21.9)	39 (24.2)	22 (27.8)	26 (22.8)	
	**Duration of last quit attempt, n (%)**						.497
		≤3 days	34 (49.3)	82 (55.0)	44 (59.5)	59 (60.2)	
		4+ days	35 (50.7)	67 (45.0)	30 (40.5)	39 (39.8)	
	Desire to quit, mean (SD)		9.25 (1.08)	9.07 (1.24)	8.87 (1.25)	8.95 (1.43)	.263
	Confidence in quitting, mean (SD)		6.49 (2.09)	6.48 (2.13)	5.72 (2.28)	6.16 (2.08)	.052
**Psychosocial variables**								
	**Health status, n (%)**						.558
		Excellent	12 (16.4)	13 (8.1)	7 (8.9)	11 (9.7)	
		Very good	26 (35.6)	63 (39.1)	26 (32.9)	40 (35.4)	
		Good	20 (27.4)	57 (35.4)	31 (39.2)	35 (31.0)	
		Fair/Poor^d^	15 (20.5)	28 (17.4)	15 (19.0)	27 (23.9)	
	Illness caused by smoking, n (%)		45 (61.6)	105 (65.2)	40 (51.3)	59 (51.8)	.069
	Spouse smokes, n (%)		20 (51.3)	40 (42.6)	23 (45.1)	31 (48.4)	.787
	1+ smokers in house, n (%)		17 (23.3)	23 (14.3)	11 (13.9)	27 (23.7)	.103
	Temptations Inventory, mean (SD)		4.00 (0.47)	3.90 (0.49)	3.90 (0.59)	3.92 (0.52)	.567
	Confidence Inventory, mean (SD)		2.88 (0.60)	2.82 (0.57)	2.67 (0.48)	2.71 (0.57)	.045
	Perceived Stress Scale, mean (SD)		6.10 (3.11)	5.90 (2.91)	6.25 (3.26)	6.89 (3.18)	.067
	CES-D Scale, mean (SD)		8.79 (5.78)	8.73 (5.27)	9.96 (6.12)	10.31 (5.81)	.082

^a^Nonparametric test (categorical) or ANOVA used.

^b^Participants could refuse to answer a question or respond “I don’t know”. Income, n=423; duration of last quit attempt, n=390; health status, n=426; illness caused by smoking, n=426; spouse smokes, n=248 (asked only among individuals with spouse).

^c^Includes part-time employment, retired, student, homemaker, and unemployed.

^d^Collapsed “Fair” and “Poor” categories due to small cell counts.

**Table 2:** Treatment utilization and smoking outcomes at 3 months by latency to target quit date (TQD) relative to website registration date.

Dependent measure			TQD, 0 days	TQD, 1-14 days	TQD, 15-28 days	TQD, 29+ days	*P* value^a^
			n=73	n=161	n=79	n=114	
**Website utilization**							
	**Logins, n (%)**						.024
		1-2	14 (19.2)	51 (31.7)	17 (21.5)	37 (32.5)	
		3-5	11 (15.1)	29 (18.0)	26 (32.9)	28 (24.6)	
		6-10	18 (24.7)	28 (17.4)	9 (11.4)	19 (16.7)	
		More than 10	30 (41.1)	53 (32.9)	27 (34.2)	30 (26.3)	
	Page views, median (interquartile range)		138 (362)	102 (198)	98 (256)	59.50 (158)	.016
	Total number minutes online, median (interquartile range)		88 (237)	62 (157)	54 (150)	43 (119)	.212
	Number of quit dates set using Quit Date Wizard, mean (SD)		2.44 (1.73)	1.95 (1.42)	1.72 (0.97)	1.57 (1.40)	.002
	Used one-to-one messaging, n (%)		23 (31.5)	38 (23.6)	16 (20.3)	15 (13.2)	.023
	Used Medication Wizard, n (%)		12 (16.4)	53 (32.9)	30 (38.0)	35 (30.7)	.025
**Other treatment utilization at 3 months (among those who made a quit attempt, n=389)** ^b^							
	Used pharmacotherapy, n (%)^c^		38 (55.9)	93 (63.7)	47 (61.8)	52 (53.1)	.350
	Used behavioral treatment, n (%)^d^		10 (14.7)	33 (22.6)	23 (30.3)	21 (21.4)	.167
	No. counseling calls completed, mean (SD)^e^		3.39 (2.72)	4.43 (2.87)	4.47 (2.58)	4.83 (3.03)	.159
**Smoking outcomes** ^f^							
	30-day ppa, n (%)		19 (26.0)	33 (20.6)	18 (22.8)	20 (17.5)	.555
	**No. quit attempts, n (%)**						.158
		0	4 (5.5)	14 (8.8)	3 (3.8)	15 (13.3)	
		1	22 (30.1)	50 (31.3)	29 (36.7)	30 (26.5)	
		2	24 (32.9)	33 (20.6)	19 (24.1)	21 (18.6)	
		3	8 (11.0)	26 (16.3)	10 (12.7)	25 (22.1)	
		4+	15 (20.5)	37 (23.1)	18 (22.8)	22 (19.5)	

^a^Nonparametric test (median; categorical) or ANOVA used.

^b^Participants could refuse to answer a question or respond “I don’t know”. Pharmacotherapy, n=388; used behavioral treatment, n=388.

^c^NRT, Zyban, Chantix.

^d^Individual counseling, group counseling, pamphlet/books, telephone counseling not through the study.

^e^Among those randomized to enhanced Internet plus telephone counseling (n=33 among TQD 0 days; n=77 among TQD 1-14 days, n=38 among TQD 15-28 days, and n=47 among TQD 29+ days).

^f^Participants were able to refuse answering a question or respond “I don’t know”. Sample sizes are follows: 30-day ppa, 426; no. quit attempts, 425.

### Study Question 4

The final logistic regression model among prospective quit date setters did not include desire to quit and confidence in quitting measures as both were unrelated to 30-day ppa. For 30-day ppa, the interaction between number of quit dates set and logins was significant (parameter estimate=–0.003, standard error=0.001, *P*=.005). Among those with high levels of website utilization (n=214; median split), 41.8% (33/79) of those who set one quit date were abstinent compared to 25.9% (35/135) of those who changed their quit date one or more times. Among those with high logins who set only one quit date and who were abstinent, the majority (60.6%, 20/33) opted to quit within 2 weeks of website registration.

### CART Analysis

**Figure 2:**
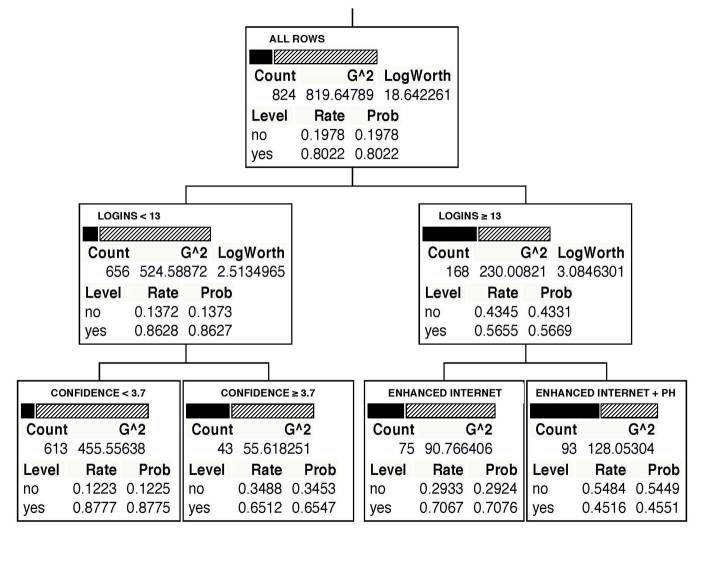
CART model predicting 30-day point prevalence abstinence at 3 months (n=824). Bars correspond to smoking status: solid=abstinent; diagonal lines=not abstinent. Count=total number of participants in subset; Level=smoking abstinence status (no/yes); Rate=relative proportion of the count in each abstinence status group; Logins=frequency of website logins during first 3 months of study; enhanced Internet and enhanced Internet + ph (telephone counseling)=treatment arms; Confidence=Confidence Inventory score.

The CART model for 30-day ppa produced a tree with splits at three nodes (Figure 2), none of which were variables associated with quit date setting or timing. The first node, representing the total sample (n=824; 1 case missing outcome data), shows the overall proportion quit (19.8%, 163/824; Level=no) compared to the proportion smoking (80.2%, 661/824; Level=yes). The first split partitioned the total sample by logins (<13 logins, 79.6% of sample; ≥13 logins, 20.4% of sample). Among those who logged in <13 times, 13.7% (90/656) were abstinent, and for individuals who logged in ≥13 times, 43.5% (73/168) were abstinent. The second split occurred among individuals who logged in <13 times and was based on the Confidence Inventory scale score. Among those with a score <3.7, 12.2% (75/613) were abstinent compared to 34.9% (15/43) among those with a score ≥3.7. The third split occurred for those who logged in ≥13 times, where the sample was divided by treatment. Among enhanced Internet participants, 29.3% (22/75) were abstinent compared to 54.8% (51/93) of enhanced Internet plus telephone counseling participants. The k-fold cross-validation results showed good agreement (similar R squared values) between the folded and overall samples.

## Discussion

### Principal Findings

In these secondary analyses of The iQUITT Study, we explored the use of an online interactive tool to set a quit date and its relationship to smoking outcomes. The majority of study participants who used the website set a quit date using the Quit Date Wizard: most set a prospective quit date, but some used it to document a quit date that had already passed. Only 9.7% (62/638) of those who used the website did not use this tool. Our a priori hypotheses were only partially supported. We did not find evidence that individuals whose first TQD was set within 2 weeks of registration differed on baseline desire to quit or motivation to quit as hypothesized, but we did find that those who set a quit date within 2 weeks of registration had higher levels of baseline self-efficacy (Confidence Inventory score) and education compared to smokers who set later quit dates. We also found that participants whose TQD occurred within the first 2 weeks of website registration exhibited higher rates of website utilization than those with later quit dates. We did not find any differences in smoking outcomes based on latency to TQD. There was an interaction between website utilization and number of TQDs set on quit rates. At low levels of website utilization, there was no difference in abstinence rates based on number of quit dates set, but at high levels of website utilization, those who set only one quit date had significantly higher quit rates than those who changed their quit date.

Overall, the CART analysis was consistent with these findings. Latency to TQD did not predict abstinence, but website utilization (logins) and baseline self-efficacy did along with treatment group. Login frequency initially split the sample, and among individuals who logged into the website more frequently, the addition of telephone counseling appeared to increase abstinence relative to enhanced Internet alone. Self-efficacy appeared to be a key variable among those with lower levels of website utilization. Among this group, higher self-efficacy scores were associated with higher quit rates. This finding is consistent with a wealth of research demonstrating the importance of self-efficacy on smoking outcomes [52-55]. The importance of logins is consistent with other Web-based trials that have reported that website utilization is an important predictor of abstinence [31,56,57]. It should also be noted that none of the metrics of motivation to quit emerged in the CART, suggesting that website utilization was a stronger predictor of abstinence than motivation to quit.

In terms of the practical relevance of these results, both traditional and exploratory analyses both point to self-efficacy and website utilization as critical components of abstinence. Findings related to Study Questions 3 and 4 suggest that individuals who set a quit date early in the course of Web-based cessation treatment are more likely to be confident about their ability to achieve cessation and that setting a TQD early on and maintaining high levels of website utilization may incur an advantage for cessation. Taken together, these results suggest that Internet cessation programs should emphasize the importance of feeling efficacious about handling specific smoking situations and engaging with treatment at the highest level possible while potentially placing less emphasis on an absolute time frame for setting a TQD (ie, within 2 weeks versus 2-4 weeks). These results are consistent with a growing body of literature demonstrating the critical importance of engagement and adherence with regard to the effectiveness of Web-based health behavior change interventions [58-66].

### Strengths and Limitations

These findings should be considered in the context of several related strengths and limitations. First, the CART analysis is a novel contribution to the literature concerning predictors of smoking abstinence. It is a powerful exploratory technique that offers an unbiased assessment of a large set of predictors and requires little input from the analyst. However, inferences based upon these analyses should be tested and replicated under controlled conditions. Second, participants were not required to set a quit date and could use the website as they desired, resulting in relatively naturalistic observations of the use of a quit date tool. Future research should examine how the use of this tool corresponds to self-reported quit attempts using other assessment methods. Third, we are unclear what to make of retrospective TQDs since current smoking status was confirmed during the baseline telephone survey. We speculate that participants may have used the Quit Date Wizard to document their most recent quit attempt or perhaps entered an erroneous date. Relative to registration, 55% of retrospective dates occurred within the week prior to study randomization, which suggests that many smokers search for cessation assistance in the early days following a quit attempt when they have returned to smoking. Qualitative methods or formal usability testing may shed light on this finding. Fourth, while the use of responder-only analyses is less conservative than intention-to-treat analyses, we feel this approach was appropriate for these exploratory analyses since imputation of missing data using an intent-to-treat (missing=smoking) approach might have obscured results. Fifth, it was not feasible to biochemically verify self-reported abstinence outcomes since this was a national sample recruited entirely via the Internet. Self-reported abstinence is a commonly accepted outcome metric in Web-based cessation trials [67-71] where misreporting of abstinence is expected to be minimal [72]. Last, we cannot rule out the possibility that low levels of website utilization were a consequence (and not cause) of relapse [73]. Studies that establish a chronological sequence of patterns of treatment utilization and relapse are needed [74].

### Conclusions

In the context of a pragmatic randomized trial of Internet and telephone treatment for cessation, the timing of a TQD was not a significant predictor of cessation outcomes. Self-efficacy and an apparent commitment to an initial TQD were the components most highly related to abstinence but only via interactions with website utilization. Increasing treatment engagement has been noted as an important area for future research in Web-based cessation studies [57,75].

## Multimedia Appendix 1

Baseline characteristics of individuals who used the Quit Date Wizard (QD) (n=576) and those who did not (n=62).

## Abbreviations

ANOVA: analysis of variance
CART: classification and regression tree
CES-D: Center for Epidemiologic Studies-Depression Scale
ph: telephone counseling
ppa: point prevalence abstinence
QD: quit date
TQD: target quit date
