# Supplementary material for: Quit Now? Quit Soon? Quit When You’re Ready? Insights About Target Quit Dates for Smoking Cessation From an Online Quit Date Tool
Source: J Med Internet Res. 2014 Feb 17;16(2):e55. doi: 10.2196/jmir.3086 (PMC3958692; doi:10.2196/jmir.3086)
Supplement: Supplementary file 1 [file jmir_v16i2e55_app1.pdf]

	QD n=576	No QD n=62	*P*-value[a]
**DEMOGRAPHIC VARIABLES[b]**			
Age, years, mean (SD)	37.09 (11.34)	34.79 (11.62)	0.131
Gender, n (%)			0.009
Female	292 (50.7)	20 (32.3)	
Race, n (%)			0.994
White	507 (88.0)	54 (87.1)	
Non-White	69 (12.0)	8 (12.9)	
Ethnicity (Hispanic), n (%)	18 (3.1)	3 (4.8)	0.731
Education, n (%)			0.151
High school or less	124 (21.5)	10 (16.1)	
Some college	269 (46.7)	37 (59.7)	
College 4+ years	183 (31.8)	15 (24.2)	
Employment, n (%)			0.761
Employed full time	409 (71.0)	45 (73.8)	
Other[c]	167 (29.0)	16 (26.2)	
Income, n (%)			0.655
Low income (<$40,000)	262 (45.8)	26 (41.9)	
High income (>$40,000)	310 (54.2)	36 (58.1)	
**SMOKING VARIABLES**			
Cigarettes per day, mean (SD)	19.63 (9.17)	20.10 (10.69)	0.708
Time to first cigarette, n (%)			0.793
Within 30 minutes	432 (75.0)	45 (72.6)	
After 30 minutes	144 (25.0)	17 (27.4)	
Duration of last quit attempt, n (%)			0.463
≤3 days	298 (56.2)	27 (50.0)	
4+ days	232 (43.8)	27 (50.0)	
Desire to quit, mean (SD)	9.07 (1.27)	9.26 (1.16)	0.263
Confidence in quitting, mean (SD)	6.28 (2.27)	6.55 (2.24)	0.369
**PSYCHOSOCIAL VARIABLES**			
Health status, n (%)			0.076
Excellent	53 (9.2)	7 (11.3)	
Very good	214 (37.2)	13 (21.0)	
Good	198 (34.4)	25 (40.3)	
Fair/Poor[d]	110 (19.1)	17 (27.4)	
Illness caused by smoking, n (%)	341 (59.3)	34 (54.8)	0.587
Spouse smokes, n (%)	157 (45.5)	24 (64.9)	0.039
1+ smokers in house, n (%)	102 (17.7)	12 (19.4)	0.883
Temptations Inventory, mean (SD)	3.89 (0.52)	3.90 (0.47)	0.810
Confidence Inventory, mean (SD)	2.82 (0.58)	2.68 (0.56)	0.075
Perceived Stress Scale, mean (SD)	6.21 (3.19)	5.89 (3.34)	0.449
CES-D Scale, Mean (SD)[e]	9.28 (5.78)	9.28 (5.77)	0.998

[a] = Non-parametric test (categorical) or ANOVA (continuous) used.
[b] = Participants were able to refuse answering a question or respond "I don't know". Sample sizes are follows: employment, 488; income, 634; duration of last quit attempt, 584; health status, 637; illness caused by smoking, 637; spouse smokes, 382 (only asked among individuals with spouse).
[c] = Includes part-time employment, retired, student, homemaker, and unemployed.
[d] = Collapsed “Fair” and “Poor” categories due to small cell counts.
[e] = CES-D: Center for Epidemiologic Studies-Depression Scale.
